# Identifying Influential Nodes in Complex Networks Based on Multiple Local Attributes and Information Entropy

**DOI:** 10.3390/e24020293

**Published:** 2022-02-18

**Authors:** Jinhua Zhang, Qishan Zhang, Ling Wu, Jinxin Zhang

**Affiliations:** 1 School of Economics and Management, Fuzhou University, Fuzhou 350108, China; zhangjin-hua@foxmail.com (J.Z.); zhangqs@fzu.edu.cn (Q.Z.); 2 College of Computer and Data Science, Fuzhou University, Fuzhou 350108, China; wuling1985@fzu.edu.cn; 3 School of Business, Hubei University, Wuhan 430062, China

**Keywords:** complex networks, influential nodes, direct influence, indirect influence, information entropy

## Abstract

Identifying influential nodes in complex networks has attracted the attention of many researchers in recent years. However, due to the high time complexity, methods based on global attributes have become unsuitable for large-scale complex networks. In addition, compared with methods considering only a single attribute, considering multiple attributes can enhance the performance of the method used. Therefore, this paper proposes a new multiple local attributes-weighted centrality (LWC) based on information entropy, combining degree and clustering coefficient; both one-step and two-step neighborhood information are considered for evaluating the influence of nodes and identifying influential nodes in complex networks. Firstly, the influence of a node in a complex network is divided into direct influence and indirect influence. The degree and clustering coefficient are selected as direct influence measures. Secondly, based on the two direct influence measures, we define two indirect influence measures: two-hop degree and two-hop clustering coefficient. Then, the information entropy is used to weight the above four influence measures, and the LWC of each node is obtained by calculating the weighted sum of these measures. Finally, all the nodes are ranked based on the value of the LWC, and the influential nodes can be identified. The proposed LWC method is applied to identify influential nodes in four real-world networks and is compared with five well-known methods. The experimental results demonstrate the good performance of the proposed method on discrimination capability and accuracy.

## 1. Introduction

Many systems in the real world can be modeled as complex networks to facilitate the study of the systems by complex network analysis. In a complex network, compared with other general nodes, some nodes can greatly affect the function of the whole network. These nodes are called influential nodes, and the quantity of these nodes is small. Ranking and identifying influential nodes in complex networks is one of the most important and basic problems in complex network research.

A variety of measures and methods have been proposed in recent decades for identifying the influential nodes of complex networks [1,2,3]. The most commonly used measures are centrality measures, such as degree centrality [4], closeness centrality [5,6], betweenness centrality [7,8], Katz centrality [9,10], and k-shell [11]. Degree centrality is the simplest metric that considers only the direct neighbors of a target node. Therefore, the degree centrality has low time complexity. However, its accuracy is also low since it ignores the global structure information of the network. Path-based centralities, such as closeness centrality, betweenness centrality, and Katz centrality can get better results. However, as global metrics, they are not suitable for large-scale networks due to their high computational complexity. Kitsak et al. [11] found that the influence of a node is highly related to the position of the node in a network, so they proposed to apply the k-shell decomposition method to identify the influential nodes of the network. However, k-shell decomposition is a coarse-grained method; the same k-shell value is assigned to a large number of nodes. Therefore, scholars have proposed a series of improved methods based on k-shell decomposition [12], such as mixed degree decomposition [13], improved neighbors’ k-core [14], neighborhood coreness [15], weighted k-shell [16], k-shell iteration factor [17], extended local k-shell sum [18], influence capability [19], hierarchical k-shell [20], integral k-shell [21], improved k-shell hybrid method [22], etc. As for the field of the search engine, there are also some useful methods, such as PageRank [23], LeaderRank [24], and Hits [25]. Other centrality measures include H-index [26,27] and several measures based on the clustering coefficient, such as local centrality [28] and clustered local-degree [29], etc. In addition, information entropy has also been used to evaluate the influence of nodes in complex networks [30,31,32,33,34].

In recent years, some scholars have made practical explorations from new perspectives. Some researchers have found that the influence of nodes includes direct local influence and indirect influence based on neighbors. Therefore, these researchers constructed influential node identification models based on direct influence and indirect influence [30,31,33,35,36]. Some other researchers have found that considering multiple attributes can achieve better node influence evaluation results than considering only a single attribute. Wang et al. [19] applied a multi-attribute ranking method based on a node’s position and neighborhood information. Yang et al. [37] applied TOPSIS to identify influential nodes and combined it with grey relational analysis to rank the importance of complex network nodes. Mo and Deng [38] adopted D-S evidence theory to comprehensively consider degree centrality, betweenness centrality, efficiency centrality, and correlation centrality for evaluating the importance of nodes in complex networks. These multi-attributes methods have proved to be promising strategies to evaluate the influence of nodes. However, there are still some limitations in existing multi-attribute methods, such as some methods being based on global attributes during the evaluation, which results in high computational complexity, unsuitable for current fast-growing social networks. In some methods, the contribution of each attribute is viewed as equal, or the weights of different attributes are set manually, which results in limited improvement in evaluating performance.

Degree and clustering coefficient are two critical attributes in node influence evaluation methods. In particular, degree and clustering coefficient belong to local attributes and have low computational complexity. Meanwhile, Liu et al. [39] deeply studied the role of neighbors on the influence of nodes and concluded that considering the one-step or two-step neighborhood of a node can achieve the best performance in most networks. Inspired by the above discussion, we propose a new multi-attribute weighted centrality based on information entropy, combining degree and clustering coefficient; both one-step and two-step neighborhood information are considered. The main contributions of this paper are summarized as follows. First, this paper considers the indirect influence of nodes and proposes a new measure of indirect influence, i.e., the two-hop clustering coefficient. Second, this paper improves the calculation formula of the two-hop degree. In the existing two-hop degree calculation, the degrees of one-hop neighbors are directly added. In our proposed two-hop degree formula, we take a more reasonable process, eliminating duplicate neighbors and then adding the neighbors of one-hop neighbors. Last but not least, this paper proposes a new multi-attribute weighted centrality with a low computational complexity O(n), as it is calculated based on four local attributes, including degree, two-hop degree, clustering coefficient, and two-hop clustering coefficient. Additionally, the entropy weighting method is exploited to weight the contributions of the four local attributes, rather than weight them equally.

The rest of this paper is organized as follows. In Section 2, relevant knowledge is introduced as a preliminary, including the six kinds of well-known centrality measures and the information entropy theory. In Section 3, the proposed multi-attribute weighted centrality is described. In Section 4, the proposed method is applied to four real world networks and compared with five well-known methods to verify the effectiveness and practicability of our proposed method. Section 5 concludes the paper.

## 2. Preliminaries

### 2.1. Typical Centrality Measures

The influence of a node can be referred to as centrality. Scholars have proposed a variety of centrality measures to evaluate and rank the influence of nodes. In this section, we briefly introduce six typical centrality measures, including degree, betweenness centrality, closeness centrality, local centrality, clustering coefficient, and clustered local degree. In the following part of this paper, new centrality measures based on the degree and clustering coefficient are proposed, and some of these centrality measures will be used as comparison methods for experiments.

**Definition** **1.** *(Degree* [4]*). The degree of node* i*, denoted as* $k_{i}$*, which is defined as*

(1) $$k_{i}=\sum_{j}^{N}a_{ij}$$

*where* N *is the node set, node* i,j∈N*, and* $a_{ij}$ *refers to the connection between node* i *and node* j*. The value of* $a_{ij}$ *is defined as* $a_{ij}=\left\{ \begin{array}{l} 1,\;node\;i\;connected\;with\;j\\ 0,\;otherwise \end{array}\right.$.

**Definition** **2.** *Betweenness centrality* [7,8]. *The betweenness centrality of node* i*, denoted as* $b_{i}$*, is defined as*

(2) $$b_{i}=\sum_{j\neq s\neq i}\frac{n_{js}(i)}{n_{js}}$$

*where* $n_{js}$ *refers to the number of all the shortest paths between node* j *and node* s*, and* $n_{js}(i)$ *denotes the number of those shortest paths which go through node* i.

**Definition** **3.** *Closeness centrality* [5,6]*. The closeness centrality of node* i*, denoted as* $c_{i}$*, is defined as*

(3) $$c_{i}=\frac{1}{\sum_{j}^{N}d_{ij}}$$

*where* $d_{ij}$ *refers to the distance between node* i *and node* j*. If there is no path between node* i *and node* j*,* $d_{ij}=\infty$*, then* $\frac{1}{d_{ij}}=0$ [5,40].

**Definition** **4.** *Local centrality* [28]*. The local centrality of node* v*, denoted as* $C_{L}(v)$*, is defined as*

(4) $$Q(u)=\sum_{w\in \Gamma (u)}N(w)$$

(5) $$C_{L}(v)=\sum_{u\in \Gamma (v)}Q(u)$$

*where* Γ(u) *is the set of the one-hop neighbors of node* u*,* Γ(v) *is the set of the one-hop neighbors of node* v*, and* N(w) *is the number of the one-hop and the two-hop neighbors of node* w.

**Definition** **5.** *Clustering coefficient* [37]*. The clustering coefficient of node* i*, denoted as* $clc_{i}$*, is defined as*

(6) $$clc_{i}=2e_{i}/(k_{i}(k_{i}-1))$$

*where* $k_{i}$ *refers to the degree of node* i*, which can be calculated as Formula (1), and* $e_{i}$ *represents the number of edges connected between the neighbors of node* i.

**Definition** **6.** *Clustered local degree* [29]*. The clustered local degree of node* i*, denoted as* $cld_{i}$*, is defined as*

(7) $$cld_{i}=(1+cc_{i})\sum_{j\in N(i)}k_{i}$$

*where* N(i) *is the set of the one-hop neighbors of node* i*,* $cc_{i}$ *is the clustering coefficient of node* i*, which can be calculated as Formula (6), and* $k_{i}$ *refers to the degree of node* i.

### 2.2. Information Entropy and Entropy Weighting Method

Information entropy is a famous concept of information theory, which was first proposed by Claude Elwood Shannon in his work “A Mathematical Theory of Communication” in 1948. It is a useful tool for measuring the uncertainty of information. In general, the more uncertain or random an event is, the more information it will contain. Shannon’s information entropy has been applied in many areas, such as data mining, statistical inference, machine learning, image processing, and so on.

According to Shannon’s information theory, the information entropy is defined as follows:

(8) $$H(X)=H(x_{1},x_{2},\ldots ,x_{n})=-\sum_{i=1}^{n}p(x_{i})\log p(x_{i})$$

where $X=\{x_{1},x_{2},\ldots ,x_{n}\}$ is a set of possible events, and $p(x_{i})$ is the probability of event $x_{i}$.

The entropy weighting method is one of the most important applications of information entropy in the field of social science. Due to its excellent performance, the entropy weighting method is widely used to determine the weights of different attributes in multi-attribute decision-making problems [41,42], and it is also widely used in node importance evaluation in complex networks. Therefore, in this paper, we also use the entropy weighting method to determine the weight of the four local attributes.

Suppose there are n attributes to be considered. The weight of attribute i, denoted as $w_{i}$, is calculated as:

(9) $$w_{i}=\frac{1-H_{i}}{\sum_{1}^{n}\left(1-H_{i}\right)}$$

where $H_{i}(i=1,2,\ldots ,n)$ is the information entropy of each attribute [19,43].

## 3. Method Description

### 3.1. Direct Influence of a Node with Respect to Local Attributes

As introduced in Section 1, the total influence of a node can be divided into direct influence and indirect influence. Direct influence is the influence of a node on the nodes directly connected to it, that is, the influence exerted on its one-hop neighbors. Considering the degree and clustering coefficient, these two indicators belong to local indicators, which are representative and are simple to calculate. In this study, we selected the degree and clustering coefficient to represent the direct influence of nodes. For the calculation of degree $k_{i}$ and clustering coefficient $clc_{i}$, see Definition 1 and Definition 5, respectively.

### 3.2. Indirect Influence of a Node on Two-Hop Attributes

The indirect influence of a node is the influence it exerts on nodes that are not directly connected to it. Liu et al. [40] have pointed out that the importance of a node is not only determined by its direct neighbors, but also depends on its multi-step neighbor information. According to their experimental results, for the sake of balancing accuracy and efficiency, considering two-step neighbor information is the best choice. Based on the one-hop neighborhood degree and clustering coefficient, we propose the two-hop degree and two-hop clustering coefficient as indirect influence measures. Wang et al. [19] have also utilized two-hop degree information in their research. However, in their two-hop degree formula, they directly sum the degree of a node’s one-hop neighbors, which ignores the overlap of different node’s neighbors. Therefore, in our two-hop degree formula, duplicate nodes are eliminated and calculated only once.

**Definition** **7.** *Two-hop degree. The two-hop degree of node* i*, denoted as* $thk_{i}$*, is defined as*

(10) $$thk_{i}=NN_{i}-NS_{i}-1$$

*where* $NN_{i}$ *is the number of two-hop neighbors of node* i*, and* $NS_{i}$ *denotes the number of same neighbors*.

**Definition** **8.** *Two-hop clustering coefficient. The two-hop clustering coefficient of node* i*, denoted as* $thclc_{i}$*, is defined as*

(11) $$thclc_{i}=\sum_{j\in N(i)}clc_{j}$$

*where* $clc_{j}$ *is the clustering coefficient of node* j*, which is defined in Definition 5;* N(i) *denotes the neighbors of node* i.

### 3.3. The Multiple Local Attributes Weighted Centrality

In this section, we propose a new multiple local attributes weighted centrality based on the above-mentioned direct influence measures and indirect influence measures for identifying influential nodes in a complex network. For the sake of brevity, we will abbreviate the multiple local attributes weighted centrality as LWC. The basic idea of the LWC is to view each node of the network as a decision scheme and each attribute of the node as an index of the decision scheme. Since different indices have different contributions to the influence of a node, we apply the entropy weight method to calculate the weight of each index; then, the influence of a node is the weighted sum of all indices. The steps of the LWC are described as follows.

Step 1: Construct the multi-attribute decision-making matrix of the node influence. For a complex network with n nodes and the node set $V=\{v_{1},v_{2},\ldots ,v_{n}\}$, the decision scheme can be expressed as $D=\{d_{1},d_{2},\ldots ,d_{n}\}$. In this paper, we adopt the above mentioned four local attributes, degree, two-hop degree, clustering coefficient, and two-hop clustering coefficient, as influence evaluation indices, so the evaluation indices can be expressed as $A=\{a_{1},a_{2},a_{3},a_{4}\}=\{k,thk,clc,thclc\}$. Then, $d_{i}(a_{j})(i=1,2,\ldots ,n;j=1,2,3,4)$ represents the jth index of node i, and the multi-attribute decision-making matrix P can be obtained as follows.

(12) $$P=\left[\begin{array}{cccc} \begin{array}{l} d_{1}(a_{1})\\ d_{2}(a_{1})\\ \ldots \\ d_{n}(a_{1}) \end{array} & \begin{array}{l} d_{1}(a_{2})\\ d_{2}(a_{2})\\ \ldots \\ d_{n}(a_{2}) \end{array} & \begin{array}{l} d_{1}(a_{3})\\ d_{2}(a_{3})\\ \ldots \\ d_{n}(a_{3}) \end{array} & \begin{array}{l} d_{1}(a_{4})\\ d_{2}(a_{4})\\ \ldots \\ d_{n}(a_{4}) \end{array} \end{array}\right]$$

Step 2: Normalize the multi-attribute decision-making matrix. Due to each index having a different dimension, the matrix P should be standardized into a normalized matrix. First, set $d_{ij}=d_{i}(a_{j})/max\{d_{1}(a_{j}),d_{2}(a_{j}),\ldots ,d_{n}(a_{j})\}$, then normalize the indices $r_{ij}=d_{ij}/\sum_{i=1}^{n}d_{ij}$, and then the normalized matrix R can be obtained as follows:

(13) $$R=\left[\begin{array}{cccc} \begin{array}{l} r_{11}\\ r_{21}\\ \ldots \\ r_{n1} \end{array} & \begin{array}{l} r_{12}\\ r_{22}\\ \ldots \\ r_{n2} \end{array} & \begin{array}{l} r_{13}\\ r_{23}\\ \ldots \\ r_{n3} \end{array} & \begin{array}{l} r_{14}\\ r_{24}\\ \ldots \\ r_{n4} \end{array} \end{array}\right]$$

Step 3: Calculate the information entropy of each evaluation index. According to Formula (8), the information entropy of index j can be calculated by

(14) $$E_{j}=-\ln{\left(n\right)}^{-1}\sum_{i=1}^{n}r_{ij}\ln r_{ij}(j=1,2,3,4)$$

Step 4: Determine the weight of each evaluation index. According to Formula (9), the weight of index j can be calculated by

(15) $$w_{j}=\frac{1-E_{j}}{\sum_{1}^{4}\left(1-E_{j}\right)}$$

Step 5: Calculate the influence of each node. According to the indices normalized in Step 2 and the weight obtained in Step 4, the LWC value of node i can be calculated by

(16) $$LWC_{i}=\sum_{j=1}^{4}w_{j}r_{ij}$$

Step 6: Rank the value of the $LWC_{i}$ in descending order to obtain the node influence ranking list.

Next, the time complexity of the LWC is analyzed. Step 1 needs to calculate degree k, clustering coefficient clc, two-hop degree thk, and two-hop clustering coefficient thclc. The complexity of degree k and clustering coefficient clc are O(n). The complexity of two-hop degree thk and two-hop clustering coefficient thclc are O(2n). Step 2 is normalization, and the complexity is O(2n). Step 3 and Step 4 apply the entropy weighting method to weight the four evaluation indices, and the complexity is O(4n). Step 5 calculates the weighted sum to obtain the LWC value, and the complexity is O(n). So, the total time complexity of the proposed LWC algorithm is O(n) + O(2n) + O(2n) + O(4n) + O(n) = O(n).

## 4. Experimental Evaluation

To verify the performance of our proposed LWC method, we compare it with five well-known ranking methods, i.e., degree [4], betweenness centrality (BC) [7,8], closeness centrality (CC) [5,6], local centrality (LocalC) [28], and clustered local degree (CLD) [29].

Four real-world networks selected from different fields are used in our experiments, which consist of sparse graphs and dense graphs: (1) football; (2) netscience; (3) email; and (4) power. The datasets of the football, netscience, and power networks are provided on http://www-personal.umich.edu/~mejn/netdata/ (accessed on 8 February 2022), and the dataset of the email network is provided on https://networkrepository.com/email-univ.php (accessed on 8 February 2022). The characteristics of these four networks are listed in Table 1, including node number |V|, edge number |E|, average degree 〈k〉, max degree $k_{max}$, and clustering coefficient ${clc}_{ave}$.

We conducted the experiments on a computer with an Intel Core i5 2.4 GHz processor and 8 GB RAM. All the codes were implemented in Python 3.9. Table 2 shows the average execution time of 100 runs for each method on the four real-world networks.

### 4.1. Discrimination Capability Analysis

Firstly, we investigate the discriminability of the ranking results obtained by different methods. The monotonicity metric is used for this purpose. The monotonicity metric M [44] is defined as follows:

(17) $$M(S)={\left[1-\frac{\sum_{s\in S}{\left|V\right|}_{s}*({\left|V\right|}_{s}-1)}{\left|V\right|*(\left|V\right|-1)}\right]}^{2}$$

where S represents the ranking list of network nodes, ${\left|V\right|}_{s}$ is the number of nodes with the same rank s of the ranking list S, and |V| represents the total number of nodes of a network. The value of M(S)∈[0,1]. The higher value of *M*, the better the discrimination capacity the ranking list has. For example, if each node has a unique rank, and the value of ${\left|V\right|}_{s}$ is 1, then M(S)=1. In this case, the ranking list has a perfect discrimination capacity.

Table 3 shows the monotonicity values of different methods on four real-world networks. In Table 3, the column M(X) refers to the monotonicity metric value calculated based on the ranking list obtained by corresponding method X on each network dataset. Bold numbers represent values that can be achieved by the method with the best monotonicity on each network. The results show that the proposed LWC has the most significant monotonicity values on the football, netscience, and email networks. On the power network, the monotonicity performance of the LWC is inferior only to the CC method. These results indicate that the proposed LWC method has excellent monotonicity capacity.

**Figure 1:**
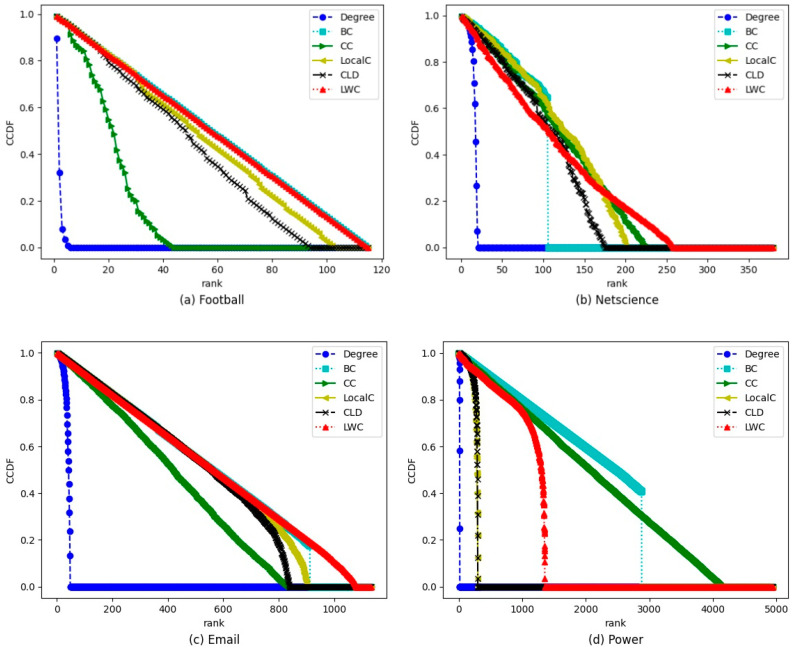
The complementary cumulative distribution function (CCDF) plots for ranking lists offered by different methods on the four real-world networks.

Secondly, in order to obtain a more precise specification of ranking distribution by different methods on the four real-world networks, the complementary cumulative distribution function (CCDF) has been plotted in addition to the monotonicity metric. Figure 1 shows the CCDF plots of different methods on the football, netscience, email, and power networks. The slower slope of the CCDF means the more distinct ranks assigned and the better performance of the ranking distribution. It can be observed in Figure 1 that the CCDF plot of the LWC decreases slowly, yielding the best monotonicity and distribution performance on the football, netscience, and email networks. For the power network, as global information methods, the BC and CC methods have a better ranking distribution performance than the LWC method. However, according to Table 2, the execution time of BC and CC are 115.32 s and 38.92 s, respectively, which is much more than the 1.39 s of the LWC method. Furthermore, if focused on the top 1000 ranked nodes, the LWC method has a similar discrimination performance to the BC and CC methods. Therefore, considering both time cost and discrimination performance, the LWC is a competitive method compared with degree, BC, CC, LocalC, and CLD.

### 4.2. Accuracy Analysis

This section investigates the accuracy of the performance of the above mentioned methods in evaluating the influence of nodes on the four real-world networks. To this end, the susceptible-infected-recovered (SIR) model is adopted to simulate the spreading influence of each node and extract the ground-truth ranking of node influence. Based on the ground-truth influence ranking, we use the Kendall coefficient, imprecision function, and Jaccard coefficient to evaluate the accuracy of different methods. Firstly, we correlate it with the ranking measure produced by each algorithm. Then, we focus on the top influential nodes to calculate the imprecision function and the Jaccard similarity coefficient. 

Researchers have proposed a large number of methods to simulate the spreading process in social networks [45]. The SIR model is one of the most popular spreading models, which has been frequently used to identify influential nodes in complex networks [46]. In the SIR model, each node has three states: susceptible, infected, or removed. To simulate the influence range of node $v_{i}$, we first set the state of node $v_{i}$ as infected, while all other nodes in the network are in a susceptible state. At each timestamp, the infected node tries to infect its neighbors, whose states are susceptible with the probability β, and then the infected node recovers with the probability γ. If a node is in a recovered state, this node is immune and cannot be infected again. Without loss of generality, the probability γ is set to 1. These infecting and recovering processes continue until a steady state is obtained. The simulation experiments are performed 100 times, and the average over these simulations is defined as a node’s influence. Finally, the ranking list based on the simulation results is marked as σ for the following accuracy analysis.

Firstly, Kendall’s τ correlation coefficient [47] is used to quantify the correlation between ranking list R obtained by each method and the ground-truth ranking list σ obtained by the SIR model. The τ coefficient is calculated as follows

(18) $$\tau (\sigma ,R)=\frac{\sum_{i<j}sgn[(x_{i}-x_{j})(y_{i}-y_{j})]}{0.5N(N-1)}$$

where sgn(x) refers to the sign function. N is the total number of nodes of the network, i.e., the length of the ranking list. $x_{i}$ and $x_{j}$ represent the ranks corresponding to node i and node j in the ranking list σ. Similarly, $y_{i}$ and $y_{j}$ represent the rank corresponding to node i and node j in ranking list R. Table 4 shows the τ correlation coefficient between the ground-truth influence ranking list σ and one produced by different ranking methods. As seen from Table 4, the correlation performance of the LWC is the best on four networks compared with the other five methods.

Since the τ coefficient considers all node pairs in the network, including a mess of insignificant nodes, the value of τ is usually not very large with the increase in the number of nodes. In practice, people are only interested in important nodes in the network. Therefore, in the next experiment, the imprecision function is adopted to quantify the accuracy performance of the most influential nodes. The imprecision function [40] is defined as

(19) $$\varepsilon (p)=1-\frac{M(p)}{Meff(p)}$$

where p is the ratio of network nodes we will investigate, p∈[0,1]. M(p) is the average spreading influence of top *pN* nodes in ranking list *R*, and Meff(p) is the average spreading influence of top *pN* nodes in ranking list σ. The smaller the value of ε(p), the more accurate the method is to identify the most influential top *pN* nodes.

**Figure 2:**
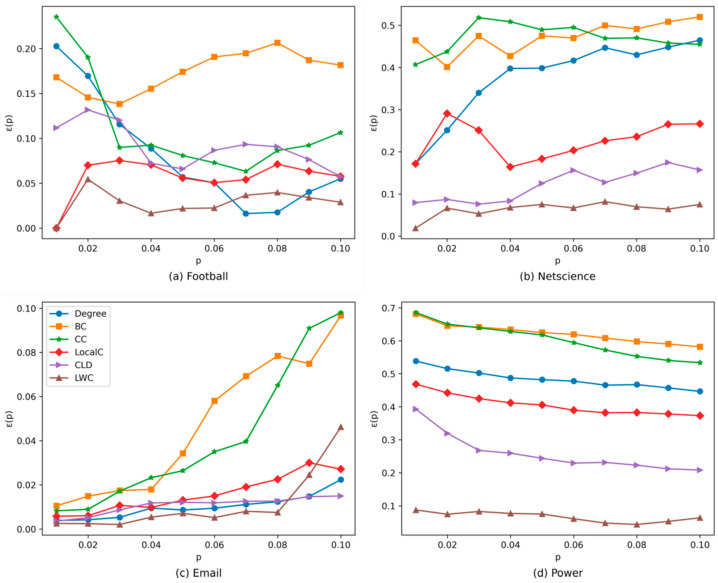
The imprecision function obtained by different methods on the four real-world networks.

Figure 2 shows the imprecision function results of different methods on the four networks. As we only focus on the most influential nodes, the value of p is set at a range from 0.01 to 0.1. It can be observed that, in the netscience and power networks, the LWC always outperforms the other methods. In the football network, only when *p* = 0.07 and 0.08 is the performance of the LWC inferior to the degree method but better than the other four methods. In the email network, the performance of the LWC is better than the other five methods, except when *p* = 0.09 and 0.1. Therefore, on the whole, the LWC method is more accurate than the other five methods.

The third metric for evaluating the accuracy of the proposed method is the Jaccard similarity coefficient [44]. Here, we consider the top *T* nodes of the ranking list σ and ranking list *R*. The Jaccard similarity coefficient *J* is obtained as

(20) $$J(T)=\frac{\left|\sigma (T)\cap R(T)\right|}{\left|\sigma (T)\cup R(T)\right|}$$

where σ(T) and R(T) are sets containing the top *T* ranked nodes of ranking list σ and ranking list *R*, respectively.

**Figure 3:**
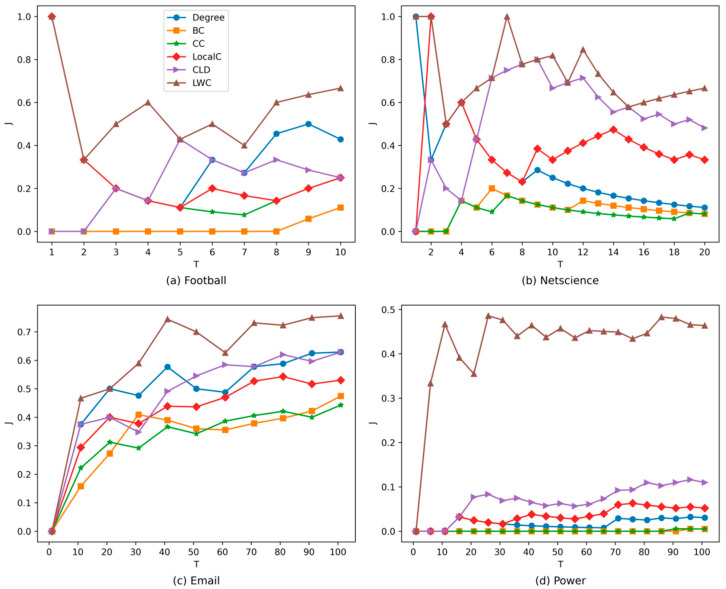
The jaccard similarity coefficient obtained by different methods on the four real-world networks.

Figure 3 shows the Jaccard coefficient of top *T* influential nodes on the football, netscience, email, and power networks. The value of *T* is set as 10, 20, 100, and 100, respectively, on the four networks. As can be seen, the LWC is again the top performer in the datasets compared with the other five methods.

## 5. Conclusions

Identifying influential nodes in a complex network is a fundamental question that has many promising applications in various fields, such as identifying super-spreaders or opinion leaders in social networks, carrying out viral marketing, controlling disease propagation, stopping the spread of rumors, accelerating information propagation in a network, and so on. However, with the booming of large-scale networks, methods based on a global attribute with high time complexity have become inapplicable. In addition, some studies have pointed out that methods considering multiple attributes can achieve better performance than methods that consider only a single attribute. Therefore, this paper proposed a low time complexity method based on four local attributes, i.e., the multiple local attributes weighted centrality (LWC), to evaluate and identify node influence in complex networks. In the LWC method, we improved the calculation formula of the two-hop degree; proposed a two-hop clustering coefficient; applied the information entropy to determine the weight of the degree, the clustering coefficient, the two-hop degree, and the two-hop clustering coefficient; and then calculated the weighted sum of these four local attributes. Compared with the degree, the betweenness centrality, the closeness centrality, the local centrality, the lustering coefficient, and the clustered local-degree methods, the experiment results on four real-world networks have shown that the proposed LWC can identify influential nodes effectively and accurately. However, there are still some challenges that need to be further overcome to improve our work. Firstly, the performance of our proposed method is better than the comparison method, but it is not very good and still has great potential for improvement. Secondly, this paper only considers the node influence in static networks. However, complex networks, in reality, sometimes change dynamically. Therefore, in future research, we will continue to try to improve the performance of the algorithm and explore dynamic network characteristics.

## Figures and Tables

**Table 1:** Specific statistical parameters of four real-world networks.

Network	|V|	|E|	〈k〉	$k_{max}$	${clc}_{ave}$
Football	115	613	10.66	12	0.403
Netscience	379	914	4.823	34	0.741
Email	1133	5451	9.622	71	0.202
Power	4941	6594	2.669	19	0.080

**Table 2:** The execution time of six methods on four real-world networks (seconds).

Network	Degree	BC	CC	LocalC	CLD	LWC
Football	0.05	0.12	0.09	0.12	0.05	0.07
Netscience	0.11	0.84	0.28	0.14	0.09	0.17
Email	0.39	8.12	2.92	0.26	0.45	0.52
Power	0.57	115.32	38.92	0.88	0.76	1.39

**Table 3:** Monotonicity performance of different methods.

Network	M(Degree)	M(BC)	M(CC)	M(LocalC)	M(CLD)	M(LWC)
Football	0.3637	1.0000	0.9488	0.9960	0.9915	1.0000
Netscience	0.7642	0.3390	0.9928	0.9887	0.9793	0.9944
Email	0.8874	0.9400	0.9988	0.9981	0.9974	0.9997
Power	0.5927	0.8319	0.9998	0.9014	0.9001	0.9653

**Table 4:** Correlation between the ranking list obtained by different methods and the ground-truth.

Network	Degree	BC	CC	LocalC	CLD	LWC
Football	0.4089	0.2801	0.3516	0.4781	0.3603	0.4931
Netscience	0.2714	0.0116	0.1835	0.3826	0.4477	0.5048
Email	0.6603	0.4755	0.5644	0.6482	0.7036	0.7217
Power	0.3569	0.1916	0.3695	0.4959	0.5639	0.5670

## Data Availability

Not applicable.
